# Can precision antibiotic prescribing help prevent the spread of carbapenem-resistant organisms in the hospital setting?

**DOI:** 10.1093/jacamr/dlad036

**Published:** 2023-03-29

**Authors:** Vasin Vasikasin, Timothy M Rawson, Alison H Holmes, Jonathan Otter

**Affiliations:** NIHR Health Protection Research Unit in Healthcare Associated Infections and Antimicrobial Resistance, Imperial College London, Hammersmith Hospital, Du Cane Road, Acton, London, W12 0NN, UK; Department of Internal Medicine, Phramongkutklao Hospital, 315 Ratchavithi Rd., Ratchadhevi, Bangkok, 10400, Thailand; NIHR Health Protection Research Unit in Healthcare Associated Infections and Antimicrobial Resistance, Imperial College London, Hammersmith Hospital, Du Cane Road, Acton, London, W12 0NN, UK; NIHR Health Protection Research Unit in Healthcare Associated Infections and Antimicrobial Resistance, Imperial College London, Hammersmith Hospital, Du Cane Road, Acton, London, W12 0NN, UK; Faculty of Health and Life Sciences, University of Liverpool, Liverpool, L69 7TX, UK; Infection Prevention and Control, Guy’s and St. Thomas’ NHS Foundation Trust, London, SE1 7EH, UK

## Abstract

The emergence of carbapenem-resistant organisms (CROs) is a significant global threat. Reduction of carbapenem consumption can decrease CROs. In the global endemic era of ESBL-producing bacteria, carbapenems are considered the treatment of choice, leading to challenge in limiting carbapenem use. This review describes the role of precision prescribing for prevention of CROs. This involves improving antibiotic selection, dosing and shortening duration. The effect of different antibiotics, dosing and duration on CRO development are explored. Available options for precision prescribing, gaps in the scientific evidence, and areas for future research are also presented.

## Introduction

Carbapenem-resistant organisms (CROs), including carbapenem-resistant Enterobacterales (CRE), carbapenem-resistant *Acinetobacter baumannii* (CRAB), and carbapenem-resistant *Pseudomonas aeruginosa* (CRPA), are increasing in incidence globally.^1^ CRE can be categorized into carbapenemase-producing CRE (CP-CRE) and non-carbapenemase-producing CRE (non-CP-CRE).^2^ CP-CRE is the most problematic among CROs due to plasmid localization allowing for horizontal gene transfer in both environmental and clinical Enterobacterales.^2,3^

The emergence of CROs is a significant global threat.^1^ Mortality following CRO infection is up to 50%, largely due to the limited treatment options.^4^ Consequently, they have been categorized by the WHO as critical priority pathogens for discovery, research and development of new antibiotics.^5^

CRO transmission frequently occurs in hospital,^6,7^ especially CRPA and CRAB.^8,9^ CRO outbreaks incur a high cost for the hospital.^10^ Cost associated with CROs is not only from direct cost such as antibiotics, contact precautions or decontamination, but also indirect cost from bed closure and missed opportunities for patient care.^10^ Several hospital infection prevention and control interventions are deployed to tackle the problem.^11–15^ These interventions include surveillance, hand hygiene, standard and transmission-based precautions, isolation, antibiotic stewardship, decolonization and environmental hygiene.

Core strategies of antibiotic stewardship include interventions to reduce inappropriate antibiotic use, antibiotic optimization, diagnostic approaches and programme measurement.^16^ Interventions focusing on reducing antibiotic consumption are effective in reducing CROs.^17^ For example, reduction of carbapenem consumption can decrease incidence of CRE,^18^ CRPA^18–20^ and CRAB.^18,21^

In the era of resistance, it is becoming difficult to restrict broad-spectrum antibiotic use. Carbapenems are considered the treatment of choice for ESBL-producing Enterobacterales (ESBL-E).^22^ The global endemic nature of ESBL-E in recent decades has led to an inevitable rise in carbapenem use.^23^

If carbapenem use is now unavoidable in many circumstances, the optimizing of antibiotic use, including careful selection of agents in these contexts, may be considered as an important intervention in local infection prevention and control strategies to minimize the development of antimicrobial resistance, particularly carbapenem resistance, and drug-resistant hospital-acquired infections.^24^ Optimizing antibiotics involves precision prescribing, allergy assessment and timely use of oral antibiotics.^16^ This review will focus on the role of precision prescribing for prevention of CROs, which is a key aspect of antibiotic stewardship. This involves improving antibiotic selection, dosing and shortening duration through the application of more individualized interventions. First, the effect of different antibiotics, dosing and duration on resistance development will be explored. We will then explore available options for antibiotic selection, dosing and duration, with an aim to prevent CROs.

## Effects of different antibiotic agents, dosing and duration on CRO emergence

**Figure 1.**
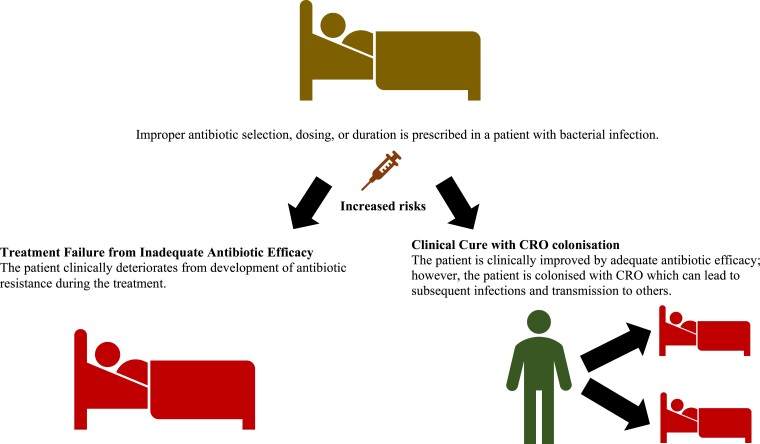
Potential antimicrobial resistance risks increased by improper antibiotic prescription.

There is emerging evidence describing the effects of different antibiotics, doses and treatment durations on resistance development. CRO emergence in this section is defined as isolation of CRO in any specimen obtained from the same individual during or after exposure to antibiotics. This does not include treatment failure caused by antibiotic resistance developing during the treatment with the specific antibiotic for the same organism, as shown in Figure 1. Efficacy and treatment failure will be discussed in the next section.

### Effects of different antibiotics on CRO emergence

Numerous efforts have been made to find and characterize the effect that individual antimicrobials exert on selection of resistance.^25^ Notably, the WHO classifies antibiotics in the essential medicines list (EML) into three groups based on risk of toxicity and resistance selection: Access, Watch and Reserve (AWaRe).^26^ For CRO, a recent meta-analysis of 349 studies by Sulis *et al*.^27^ demonstrated that carbapenem use at both individual and population levels is the strongest risk factor for CRO colonization/infection. CRPA colonization/infection is most strongly associated with carbapenem use (OR 3.2; 95% CI 2.5–4.2), followed by CRE (OR 2.5; 95% CI 2.2–2.7) and CRAB (OR 2.2; 95% CI 1.8–2.6), respectively.

#### Effects of different antibiotic classes

Identification of antibiotics with the least selective pressure for CRO is important as it can help guide alternative, carbapenem-sparing regimens. The meta-analysis by Sulis *et al*.^27^ defined that other significant antibiotics associated with CRO include lincosamides (OR 2.4 for CRE), polymyxin (OR 2.4 for CRE), tigecycline (OR 2.4 for CRE), linezolid (OR 2.1 for CRE), fourth-generation cephalosporins (OR 1.7–2.0 for CRO), glycopeptides (OR 1.5–1.9 for CRO), daptomycin (OR 1.8 for CRE), macrolides (OR 1.6 for CRE), fluoroquinolones (OR 1.4–1.9 for CRO) and piperacillin/tazobactam (OR 1.3–1.5 for CRO). It should be noted that polymyxin and tigecycline, reserved for the treatment of CRO, are also amongst the strongest risk factors of CRE colonization/infection. However, these associations should be interpreted with caution as it is difficult to control all the confounding factors influencing antibiotic selection.^25^ For example, glycopeptides do not have activity against most Gram-negative pathogens, but are often used in combination with broad spectrum β-lactams, such as carbapenems, as empirical treatment in hospitalized patients.^28^ Moreover, these associations are usually derived from population-level instead of patient-level data, disregarding the complexity of clinical parameters, epidemiological factors and antibiotic exposure in each patient.^29,30^ Because this complexity may be too difficult for classical statistical analysis, machine learning is expected to be used in the future to define antibiotic pressure in individual patients.^31^ Machine learning was successful in estimating antibiotic exposure and ESBL-E colonization in a recent study.^32^ The random forest model derived from machine learning could rank antibiotics, whether used as monotherapy, used in combination or used sequentially, based on the measurement of error in predicting ESBL-E colonization. However, there is currently no machine learning model to estimate the impact of antibiotic exposure on CRO colonization/infection. This model may be needed to understand the complex interaction between antibiotic utilization and development of CRO from patient-level data.

#### Effects of different carbapenem agents

Mechanisms of carbapenem resistance are different amongst CROs. For CRE, the selection of carbapenemase is the most important mechanism, whereas for CRPA, permeability changes (e.g. OprD) and multidrug efflux pumps (e.g. MexA-MexB-OprM) are usually the determining factors.

Carbapenem agents consist of imipenem, meropenem, ertapenem, doripenem, panipenem, biapenem, razupenem, tebipenem and faropenem. Individual carbapenems exert variable selection pressure on CRO. Selecting a carbapenem with least resistance pressure has been proposed as a potential method to reduce the occurrence of CROs.^33^ Any carbapenem use can aggravate the occurrence of CRE, with ertapenem the least selective within the group.^33^ On the contrary, ertapenem use, unlike other carbapenems, is not associated with the emergence of CRPA and CRAB.^34–37^ However, it should be noted that a strategy of replacing group 2 carbapenems with ertapenem failed in reducing CROs in three studies of carbapenem stewardship.^38^ The mandatory use of ertapenem for ESBL-E infections did not lead to a post-intervention reduction in CRAB and CRPA.^38^

#### Effect of different antibiotic combinations

Antibiotic combination therapy has also been proposed as a strategy to suppress the emergence of resistance. Combination therapy is an effective strategy to suppress the emergence of resistance in mycobacterial infections such as tuberculosis and leprosy.^39^ These slow-growing bacteria are capable of producing subpopulations of non-replicating or slowly growing cells in response to antimicrobials, also called persister subpopulations, resulting in drug tolerance.^40^ Therefore, treatment of mycobacteria consists of exceptionally long courses of antimycobacterial combinations. The mechanism of combination antimycobacterial therapy is thought to occur by resistance suppression, i.e. the first drug kills mutants resistant to the second drug, while the second drug kills those resistant to the first drug.^39^

For pyogenic bacteria that have more rapid growth, the possible benefits of antibiotic combination to prevent resistance emergence are found in *P. aeruginosa*.^41–44^ Various preclinical studies in *P. aeruginosa* infection found benefit in preventing resistance emergence in antibiotic combinations of β-lactams with aminoglycosides or fluoroquinolones.^41–44^ Early clinical studies suggested that a β-lactam in combination with an aminoglycoside could delay resistance development compared with monotherapy.^45^ However, several subsequent clinical studies and meta-analyses in *P. aeruginosa* infection have failed to demonstrate any benefit of combination treatment to the emergence of resistance.^42^ Because of the increased toxicity, continuous aminoglycoside combination therapy is not recommended for suppressing resistance.^42,46^ There are no clinical data evaluating fluoroquinolones in combination with β-lactams for this specific purpose.

### Effects of different antibiotic dosing on CRO emergence

Drug exposure can have a significant impact on resistance development. *In vitro* experiments demonstrate low resistance selective pressure occurs when drug exposure is at relatively low or high concentrations, forming an ‘inverted U’ relationship.^47^ Antibiotic concentrations achieved *in vivo* using recommended doses for clinical cure are associated with greater selective pressure for resistance.^48^ For carbapenems, this phenomenon is found in experiments of *P. aeruginosa* and may explain resistance development during therapy.^49–52^ A meta-analysis of 28 studies found that CRPA developed in 35% of patients with *P. aeruginosa* infections during carbapenem treatment without resulting in treatment failure.^53^ Therefore, increased target concentration of carbapenems may arguably be justified in *P. aeruginosa* infection to suppress resistance, but not to improve clinical outcomes.^54^

On the other hand, for Enterobacterales, the concentration for maximal killing is the same as for suppression of carbapenem resistance.^55^ A study found that the percentage of the dosing interval for drug concentration to remain above MIC (*fT*_>MIC_) for the maximal killing effect of razupenem for Enterobacterales was 63%–92%, but CRE rarely occurred at >70%.^55^ In clinical practice, CRE and CRAB rarely emerged during carbapenem treatment (<1%).^53^ Therefore, increased target concentration may not be needed in general cases of Enterobacterales and *Acinetobacter* spp. infection.

Although optimizing antimicrobial dosing to suppress resistance may seem plausible, it has potential consequences. Antibiotics can transform intestinal flora into a reservoir of antibiotic-resistant organisms, also called the gut resistome.^56^ Intestinal carriage is an important source of transmission.^57,58^ Resistance can occur even with antibiotics with minimal bile excretion into the intestine, such as cefotaxime.^59,60^ A recent study found that increasing ciprofloxacin pharmacokinetic/pharmacodynamic (PK/PD) target attainment did not reduce the chance of resistance emergence.^61^ Moreover, higher ceftriaxone PK/PD indices were associated with increased amplification of resistant genes.^62^ In clinical practice, resistance commonly emerges away from the site of the primary infection.^63,64^ Therefore, serum antibiotic concentrations should be aimed at maximal treatment efficacy and minimal toxicity. For most infections, using current PK/PD approaches, it is difficult to include resistance suppression in the treatment plan.^65^

### Effect of antibiotic duration on CRO emergence

Historically, the misconception that shortening antibiotic duration can lead to resistance emergence was held by clinicians and the general population.^66,67^ Evidence from experimental models and subsequent clinical studies has demonstrated that shortening duration can reduce resistance emergence.^68–70^ Regarding CROs, prolonged duration of many antibiotics is associated with resistance acquisition. Prolonged duration of piperacillin/tazobactam and aminoglycosides was associated with subsequent CRO acquisition (OR = 1.13 and 1.62, respectively).^71^ Prolonged duration of β-lactams with β-lactamase inhibitors (BL-BI), or carbapenems and fluoroquinolones was associated with subsequent CRE infection (OR 1.15 and 1.02 per day increase, respectively).^72^ Prolonged duration of fluoroquinolones, broad-spectrum cephalosporins and carbapenems was associated with CRAB infection [risk ratio (RR) 81.2, 31.3, and 112.1, respectively].^73^

Although optimizing the duration of antibiotic is one of the strategies of antibiotic stewardship, not many studies specifically evaluate its effectiveness in reducing CRO acquisition. A resistome study nested in a clinical trial found that shortening antibiotic duration from 14 to 7 days did not result in a decreased quantity of carbapenem resistance genes in gut microbiota.^74^ However, another study showed that early carbapenem de-escalation was associated with a shorter duration of carbapenem usage by 2 days and lower incidence of CRAB acquisition.^75^ A reduction in CRE and CRPA was also observed in this study, although this was not statistically significant.

In conclusion, the current evidence suggests that the best option for minimizing CRO emergence is selecting alternative agents in place of carbapenems. If carbapenem usage is unavoidable, options to reduce antibiotic resistance are dosing carbapenem therapy to achieve defined PK/PD targets for efficacy and prescribing the shortest effective duration.

## Carbapenem-sparing options for the treatment of ESBL-E

Optimal antibiotic selection involves choosing the most appropriate antibiotic to achieve efficacy in managing infections whilst minimizing adverse events associated with antibiotic use, including development of antibiotic resistance.^76^ Because carbapenem use is amongst the strongest risk factors for CROs, research has focused on carbapenem-sparing strategies for the treatment of ESBL-E.^77–83^ These strategies include combination antibiotic regimens, evaluation of other potential antibiotics, and selecting ertapenem as a carbapenem agent with the narrowest spectrum.

### Combination therapy

The main benefit of combination therapy for ESBL-E is likely to be promoting antibiotic synergy and broadening the spectrum of coverage.

For targeted therapy, combination antimicrobial therapy was investigated clinically for synergistic effects based on organism *in vitro* results. Apart from the standard combination of BL-BIs, the most frequently described combination antibiotics for ESBL-E are aminoglycosides, in combination with β-lactams, such as cefepime or piperacillin/tazobactam.^84–86^ Several studies have been unable to demonstrate evidence of improved clinical outcomes of combination therapy compared with β-lactam monotherapy for ESBL-E.^87,88^ Instead, increased toxicity may be observed in the combination group.^87^ Moreover, a recent study found that some *Escherichia coli* strains can exhibit *in vitro* antagonistic effects between aminoglycosides and β-lactam combinations.^89^

Another proposed combination option is between β-lactams, mostly between a cephalosporin and a BL-BI. A study found that an oral third-generation cephalosporin, cefixime, in combination with clavulanate can effectively treat patients with ESBL-E urinary tract infection.^90^ Another study reported clinical cure in 10 patients with ESBL-E urinary tract infection treated with ceftibuten and clavulanate.^91^ Several clinical trials are evaluating cephalosporins in combination with β-lactamase inhibitors. It should be noted that all ongoing trials are being conducted in patients with mild infections without bacteraemia.^92^

For empirical treatment, evidence from meta-analysis of six studies found that appropriate empirical antibiotic therapy, i.e. given within 24 h of initial culture, is associated with improved survival in patients with ESBL-E bacteraemia (adjusted OR = 2.03, *P* = 0.04).^93^ As combination therapy has been found to broaden the spectrum of coverage, a study also confirmed that empirical combination therapy could improve appropriateness for ESBL-E and was associated with survival.^94^

### Non-carbapenem antibiotics

For urinary tract infections without bacteraemia, several carbapenem-sparing antibiotic options for ESBL-E are available. These options include ciprofloxacin, levofloxacin and trimethoprim/sulfamethoxazole.^46^ Nitrofurantoin, piperacillin/tazobactam, amoxicillin/clavulanate and cefepime may also be used in cases of cystitis. However, for ESBL-E infection outside the urinary tract, carbapenems are the drug of choice.^46^ Potential antibiotics as alternatives for carbapenems include piperacillin/tazobactam, cephamycins, aminoglycosides and temocillin.

Broader-spectrum antibiotics than carbapenems such as tigecycline, IV fosfomycin, or newer β-lactams, such as ceftolozane/tazobactam, ceftazidime/avibactam, or cefiderocol, can be potential carbapenem-sparing options for ESBL-E.^95^ Large volumes of carbapenem consumption can be substituted by these potential agents. However, they also have greater potency to create a selective advantage for bacteria resistant to carbapenems.^96^ Several panels uniformly recommended that they should be reserved for XDR pathogens, such as CROs.^46,97–99^ Therefore, broader-spectrum antibiotics will not be discussed here.

The most extensively studied antibiotic as a carbapenem-sparing option is piperacillin/tazobactam,^78,100^ because of the high percentage of *in vitro* susceptibility in ESBL-E.^79^ However, *in vivo* susceptibility to piperacillin/tazobactam can be different from *in vitro* because of an inoculum effect. An inoculum effect is the dramatic increase of MIC due to increased bacterial load despite the initial susceptibility.^100^ Because the results from several meta-analyses and retrospective studies found conflicting results for the efficacy of piperacillin/tazobactam for the treatment of ESBL-E bacteraemia,^101,102^ a multicentre, randomized clinical trial called MERINO was conducted to evaluate the efficacy of bolus infusion of piperacillin/tazobactam 4.5 g q6h compared with meropenem 1 g q8h in patients with ceftriaxone-non-susceptible *E. coli* or *Klebsiella pneumoniae* bacteraemia.^103^ The study found that the definitive treatment with piperacillin/tazobactam did not result in a non-inferior mortality, therefore it was concluded that use of piperacillin/tazobactam was not supported. *Post hoc* analysis showed that some bacteria harbour narrow-spectrum oxacillinase (OXA) genes, which caused a major error in piperacillin/tazobactam susceptibility interpretation used in the trial.^104^ Therefore, many patients allocated to the piperacillin/tazobactam arm actually had bacteraemia resistant to piperacillin/tazobactam. The question still remains whether the extended infusion of piperacillin/tazobactam is comparable to carbapenems.^79^ There is an ongoing trial in Israel, Canada, and Italy evaluating this issue.^105^

Cephamycins, such as cefoxitin or cefotetan, may be used against ESBL-E; however, AmpC producers have unfavourable susceptibility to these.^81,106^ They may be an alternative to carbapenems only for non-severe infections and should be used at high dose and continuous infusion.^79,100,107^

Aminoglycosides may be used against ESBL-E bacteraemia. In a retrospective cohort, INCREMENT, differences of 30 day mortality between patients treated with meropenem and aminoglycosides were not observed.^108^ However, renal toxicity was common.^108^ Another retrospective study in patients with bacteraemia from urinary tract infection found that aminoglycosides were non-inferior to carbapenem or piperacillin/tazobactam in mortality, without difference in acute kidney injury.^109^ However, the subgroup analysis comparing aminoglycosides and carbapenems was not reported.

Temocillin is a β-lactamase-resistant penicillin, which was marketed in the UK in the 1980s.^110^ It was again relaunched in the 2010s in the UK and some European countries to combat drug-resistant organisms.^111^ A retrospective study found that the clinical cure rate was 93%, 83% and 100% in patients with ESBL-E urinary tract infection, bloodstream infection and hospital-acquired pneumonia, respectively.^112^ Currently, there is an ongoing clinical trial evaluating the efficacy of temocillin for ESBL-E bacteraemia.^113^

### Ertapenem

Ertapenem is an carbapenem with activity against ESBL-E but has only weak activity against *Pseudomonas* and *Acinetobacter* spp.^114^ It is marketed for use in severe community-acquired infections, where *Pseudomonas* and *Acinetobacter* spp. are unlikely, such as intra-abdominal infections, community-acquired pneumonia, acute pelvic infection, skin and soft tissue infections and complicated urinary infections. Because of the limited data on ertapenem efficacy as empirical treatment of severe ESBL-E infections, it is not recommended for patients with bacteraemia and severe infection, especially septic shock.^99^ A recent retrospective propensity score matching study in patients with ESBL-E bacteraemia reported no difference between ertapenem and other carbapenems in mortality, even in a subgroup of patients with septic shock.^115^

## Carbapenem dosing

The aim of antibiotic dosing is to keep antibiotic levels within the therapeutic window. Suboptimal levels of antibiotics are not only associated with treatment failure, but also with the emergence of resistance.^116–118^ On the other hand, supratherapeutic levels may lead to toxicity. Therefore, maintaining serum concentrations within therapeutic range is crucial.

There are many approaches for antibiotic optimization to maintain antibiotic concentrations within the therapeutic window. The most frequently described methods are the antibiotic nomogram and therapeutic drug monitoring (TDM).^24^

### Nomogram

The concept of a nomogram, sometimes called a population model or *a priori* dosing method, was introduced shortly after the discovery of antibiotics.^119,120^ This method usually relies on simple clinical parameters such as renal function and body weight.^121–123^ Some software can take more variables into account, such as gender, height, weight, age and serum creatinine.^120,124^ Compared with clinician-guided dosing, this method can improve target attainment of antibiotics such as vancomycin.^125,126^

Regarding carbapenems, three studies developed a dosing nomogram for continuously infused meropenem based on creatinine clearance (CL_CR_) of patients.^122,127,128^ All studies found that a standard daily dose of 3 g meropenem is sufficient for target attainment in patients with normal renal function and susceptible organisms. However, it remains unclear whether nomogram-based dosing would increase the frequency of achieving β-lactam target concentrations.^123^ Moreover, this method is aimed at achieving a predefined PK target, which may be different between patients.^129^

### TDM

TDM involves measuring antibiotic levels in blood, or in other biological fluids, which can be linked to antibiotic levels in blood or at the site of infection to personalize dosing.^130,131^ It is commonly employed for drugs with a narrow therapeutic index, e.g. vancomycin and aminoglycosides. TDM for vancomycin and aminoglycosides is recommended in standard guidelines for antibiotic prescribing.^16^ For antibiotics with wider therapeutic indices, e.g. β-lactams, TDM is recommended in critically ill patients because of the proven benefits of achieving target plasma drug levels.^65,132,133^ Achieving target plasma drug levels is associated with improved survival.^134,135^ However, the direct impact of TDM in improving clinical outcomes has not been shown.^132^ Despite these recommendations, TDM for β-lactam therapy is not routinely available in most institutions. In a survey in Europe, less than 3% reported TDM for β-lactams.^136^ Another survey in the USA showed that only 8% of 39 hospitals with dedicated infectious disease pharmacists had TDM for β-lactams.^137^

#### Drug assays

Different drug assays are being used routinely to measure serum levels clinically, or at the sites of infections for research purposes. Current TDM methods for antibiotics are immunoassays, chromatographic assays and biosensors.

##### Immunoassays

For widely established aminoglycosides and vancomycin levels, immunoassays are commonly used.^138^ Most immunoassays are fast, relatively inexpensive and available commercially.^139^ However, most commercially available assays cannot differentiate between bound and unbound drugs.^140,141^ Moreover, there can be differences between manufacturers.^142^

Recently, lateral flow assays (LFAs) were used to detect antimicrobials in clinical specimens. These assays are less expensive and can be a point-of-care test. Traditional LFAs are used for qualitative analysis. Recently, there has been improvements in affordable and compact detection devices, such as smartphone cameras, to support analysis and quantification of immunochromatographic results.^143^ This trend has led to the development of quantitative LFAs.

Quantitative LFAs have been evaluated as a tool for TDM for large molecules such as immunosuppressive drugs in serum.^144^ However, detection of smaller molecules such as antibiotics is more challenging because of the lack of immunogenicity. Therefore, LFAs for antibiotics often use a competitive technique instead of the common sandwich technique. The competitive technique has a major advantage in its low lower limit of quantitation (LLOQ). Therefore, the competitive technique is commonly used for detection of residual antibiotics in animal products.^145,146^ The major limitation of this technique is that it usually has a low upper limit of quantitation (ULOQ), making quantification of drug concentration within a clinically relevant range difficult. Newer techniques may increase the ULOQ. For example, a recently tested LFA for tenofovir TDM in clinical specimens has the ULOQ of as high as 100 mg/L.^147,148^

##### Chromatographic methods

For antibiotics such as β-lactams, commercial TDM assays are not available.^149^ TDM for β-lactams is usually conducted in plasma.^150–153^ Because of the unreliability of immunoassay, chromatographic methods are frequently used. The mostly commonly used method is liquid chromatography (LC).^131,154^ Detectors for chromatography also vary between laboratories, ranging from simple detectors such as ultraviolet (UV) to mass spectrometry (MS).^65,131^ MS has the highest specificity, although it is more expensive and requires time-consuming optimization.

Because of the sophistication, the adoption of β-lactam TDM is limited to large medical centres. Moreover, the turnaround time is usually prolonged.^155^ Although chromatography-based methods have a relatively short run time at 3–30 min,^156^ the typical laboratory workflows increase the average turnaround time to 18–24 h.^157^ In a study in real-world practice, this number was as high as 4 days,^158^ making prompt dose adjustment for critically ill patients challenging.

##### Biosensors

A biosensor is a device that transforms the interaction between antibiotics and bioreceptors into a quantifiable signal.^131^ Biosensors can measure serum levels from collected blood specimens or directly from patients. They can be minimally invasive and wearable, supporting the measurement of other biological fluids, which can be linked to the sites of infections. Biosensors can be classified according to their detection method, sensing mechanism, functionality or degree of invasiveness.^131^ Common antibiotic detection methods include optical and electrochemical sensors.^131,159^

Optical sensors are the most common method. These devices can measure the levels directly from the blood without using chromatography. They can be portable, offer high sensitivity and fast turnaround time.^131,160–162^ Their main limitations are the lower specificity from the background signal and the inability to be used as a wearable biosensor.

Electrochemical sensors can work with small sample volume and can be miniaturized to allow on-site monitoring.^159^ Bioreceptors for this type of sensor include antibodies,^163^ enzymes^163,164^ and aptamers.^165^ Their limitations are the lower specificity from non-specific binding and the low ULOQ for some methods and high LLOQ for others.

### Dose-adjustment strategies

After receiving the results of serum drug levels, several different dose-adjustment strategies are being implemented. These strategies include dose adjustment made specific to the intervention, dosing nomograms and software for dose optimization.^166^

#### Dose adjustment made specific to intervention

Specific intervention is the least sophisticated method. Dosing adaptation is usually simplified according to the difference between the targets and the levels measured. For example, some studies increased the frequency of the same dose by 25%–50% when the concentration was below the target and reduced the dose by 50% when the concentration was above 10 times the expected dose.^167,168^ Some studies increased the dose based on the degree of the differences. For example, one study suggested increasing antibiotic dose by one step if the levels were within 50%–100% of the target, or two steps if the levels were within 10%–50% of the target.^169^

Because some studies found that physicians may not adhere to consulting pharmacy service advice for dose adjustment,^158,170^ this may be the most feasible method for adoption in general practice. However, it is the least reliable method to attain the target levels.

#### Dosing nomogram

Dosing nomograms are well described and validated for aminoglycosides and vancomycin. However, they cannot be used in critically ill patients as most PK data are derived from non-critically ill patients.^171^ Nomograms are also available for β-lactams but they have not been validated.^127^

#### Software for dose optimization

Software for dose optimization is designed for more accurate dose adjustment. It can be categorized into two methods: linear regression models; and models that incorporate Bayesian forecasting or artificial intelligence.^129^

Linear regression (one-compartment model) is the least complicated method, which uses an algorithm to calculate a drug clearance rate from antibiotic levels derived from two different times. This method performs better than using a nomogram but still does not include other variables from the patients.^172^

Because of the limitations of the linear regression model, most software now uses real-time Bayesian forecasting. This method combines the population model (*a priori*) with the current PK information (*a posteriori*) to suggest the dose and the expected PK results after the adjustment.^120^ Many validated software programmes are available, both commercially and for free.^173–175^ This method can suggest the dose adjustment even from a single measured drug level. Moreover, it can calculate even after the first few doses before the steady-state concentration. These programmes demonstrated higher PK/PD target attainment for meropenem dosing.^176^ However, it failed to achieve target attainment for dose adjustment based on the concentration in a recent study.^177^

## Optimal carbapenem duration

Several studies have been focused on defining the least non-inferior treatment duration in various sites of common Gram-negative infection, including bacteraemia, urinary tract infection, intra-abdominal infections and pneumonia.

### Bacteraemia

For uncomplicated Gram-negative bacteraemia, three randomized controlled trials including a total of 1365 patients, 11% of whom had ESBL-E as the responsible pathogens, found that a 7 day duration was non-inferior to a 14 day duration.^178–180^ The notable exclusion criteria for uncomplicated bacteraemia were uncontrolled source of infections, site of infection requiring prolonged antibiotic course, severely immunocompromised patients, CRO infection and polymicrobial bacteraemia.

### Urinary tract infections

For urinary tract infections, a recent meta-analysis of treatment in pyelonephritis showed that a short course of antibiotic (≤7 days), regardless of antibiotic class, resulted in higher clinical cure rate with no significant difference in clinical failure.^181^ However, longer duration may be required for complicated pyelonephritis, i.e. patients with urogenital abnormalities. According to a meta-analysis of only studies including more than 20% with complicated infection, short duration had significantly higher microbiological failure.^182^ There is no clinical trial including only patients with complicated infection. However, two retrospective studies in patients with ESBL-E complicated urinary tract infection found no difference in mortality or recurrent infection in patients with ≤7 day duration of antibiotic.^183,184^

### Intra-abdominal infections

For intra-abdominal infections, a randomized controlled trial in patients with post-operative intra-abdominal infection found that 8 day duration was equivalent to 15 day duration in terms of mortality and length of stay.^185^ ESBL-E was responsible for about half of isolated pathogens. Another randomized controlled trial in patients with intra-abdominal infection and adequate source control found that 4 day duration was comparable to antibiotic discontinuation after clinical resolution in term of mortality, recurrent infection or surgical site infection.^186^

### Ventilator-associated pneumonia

For ventilator-associated pneumonia, which is commonly caused by Gram-negative pathogens, a randomized controlled trial found that 8 day duration was non-inferior to 15 day duration in terms of mortality or recurrent infection. However, subgroup analysis found that patients with non-fermenting Gram-negative infection had a significantly higher rate of recurrent infection in the 8 day arm.^70^ Another randomized controlled trial also found that 8 day duration was non-inferior to 15 day duration in terms of clinical cure and mortality, but also found a significantly higher rate of infection in the 8 day arm.^187^

## Conclusions

In the era of resistance, it is becoming difficult to restrict broad-spectrum antibiotic use. Precision prescribing, involving selection, dosing and duration of antibiotic, may help prevent CROs and associated hospital-acquired infections, and play a key role in infection prevention and control strategies. Future work must focus on improving precision antibiotic prescribing, not only to improve patient outcomes and reduce toxicity, but also to prevent resistance.

Current evidence suggests that the best option is selecting alternative agents to carbapenem with comparable efficacy. More work is required to investigate the effect of different carbapenem-sparing treatment options on CRO emergence, and their efficacy against ESBL-E as empirical and targeted treatment.

If carbapenems are unavoidable, the options include the improved dosing of the carbapenems, aiming to achieve defined PK/PD targets for efficacy, and prescribing shortest effective duration. However, to achieve target levels for minimal resistance development and maximal treatment outcome, innovation and new technological solutions are needed to provide rapid antibiotic concentrations for prompt dose adjustment.
